# Real-time botnet detection on large network bandwidths using machine learning

**DOI:** 10.1038/s41598-023-31260-0

**Published:** 2023-03-15

**Authors:** Javier Velasco-Mata, Víctor González-Castro, Eduardo Fidalgo, Enrique Alegre

**Affiliations:** 1 Department of Electrical Systems and Automation Engineering, Universidad de León, 24071 León, Spain; 2 Researcher at the Spanish National Cybersecurity Institute (INCIBE), 24005 León, Spain

**Keywords:** Computational science, Computer science

## Abstract

Botnets are one of the most harmful cyberthreats, that can perform many types of cyberattacks and cause billionaire losses to the global economy. Nowadays, vast amounts of network traffic are generated every second, hence manual analysis is impossible. To be effective, automatic botnet detection should be done as fast as possible, but carrying this out is difficult in large bandwidths. To handle this problem, we propose an approach that is capable of carrying out an ultra-fast network analysis (i.e. on windows of one second), without a significant loss in the F1-score. We compared our model with other three literature proposals, and achieved the best performance: an F1 score of 0.926 with a processing time of 0.007 ms per sample. We also assessed the robustness of our model on saturated networks and on large bandwidths. In particular, our model is capable of working on networks with a saturation of 10% of packet loss, and we estimated the number of CPU cores needed to analyze traffic on three bandwidth sizes. Our results suggest that using commercial-grade cores of 2.4 GHz, our approach would only need four cores for bandwidths of 100 Mbps and 1 Gbps, and 19 cores on 10 Gbps networks.

## Introduction

Over the last years, botnets have gained more and more presence on the Internet: in their 2020 report, the FBI’s Internet Crime Complaint Center (IC3) recorded more than $4.1 billion in losses related to cybercrime, suffered both by individuals and by companies^1^. A year later, the recorded losses ascended to $6.9 billion^2^. Botnets are one of the main concerns since they allow expanding online attacks such as Distributed Denial of Service (DDoS)^3^, Spam campaigns^4^, espionage^5^, Phishing^6^ and Ransomware^7^. This raises the necessity of developing botnet countermeasures.

Traffic analyzers can use different approaches: the first ones were based on recognizing known patterns inside the payloads of traffic^8,9^, but their main downside is that there exist obfuscation techniques to bypass these tools’ analyses^10^. Recent proposals that use Machine Learning (ML) techniques^11^ assume that botnet traffic reflects behavior of its related malware. While ML classifiers achieved great detection rates for botnets^12^, little work has been done on adapting these methods to high-demanding environments. In Industry 4.0^13^, it is necessary to carry out a quick detection of potential threats, ideally, in real time or close to real time. Current studies on Software Defined Networks (SDN) measured the hardware requirements to detect botnets^14–16^, but they were limited to mobile 5G networks. In this work we adopt a wider research line giving insights on the computer requirements to detect botnets on the standards TCP and UDP on any type of traffic networks that support these protocols, with bandwidths up to 10 Gbps. The main contributions of this paper are:

- A high-capacity approach to classify the network traffic in large bandwidths (tested up to 10 Gbps), in comparison to the state-of-the-art methods, as far as we know from our research on current literature. As a disclaimer, we refer to methods that analyze the traffic derived from TCP and UDP based communications between machines, not to methods based on DNS queries or on any kind of blacklists. The novelty of our approach is that it can work on time windows of just one second and yield the analysis of that frame in the next 1 to 2 seconds thanks to the use of only four features whose computation is very quick. Additionally, we also assess the performance of our model on saturated networks.
- Experimental evidence on why an incorrect use of the network IPs as traffic features is counterproductive.
- A novel study on the hardware required to implement our approach and achieve real-time detection. We estimate the number of CPU cores necessary to process network bandwidths of 100 Mbps, 1 Gbps and 10 Gbps, assuming that the cores work with a frequency of 2.4 GHz.

As the classifier for our approach, we selected a Decision Tree, following the results of our previous work^17^, in which we compared the computational costs of four different models – Decision Tree (DT), Random Forest (RF), k-Nearest Neighbors (kNN) and Support Vector Machines (SVM)–, and realized that DT was the most efficient among them.

**Figure 1:**
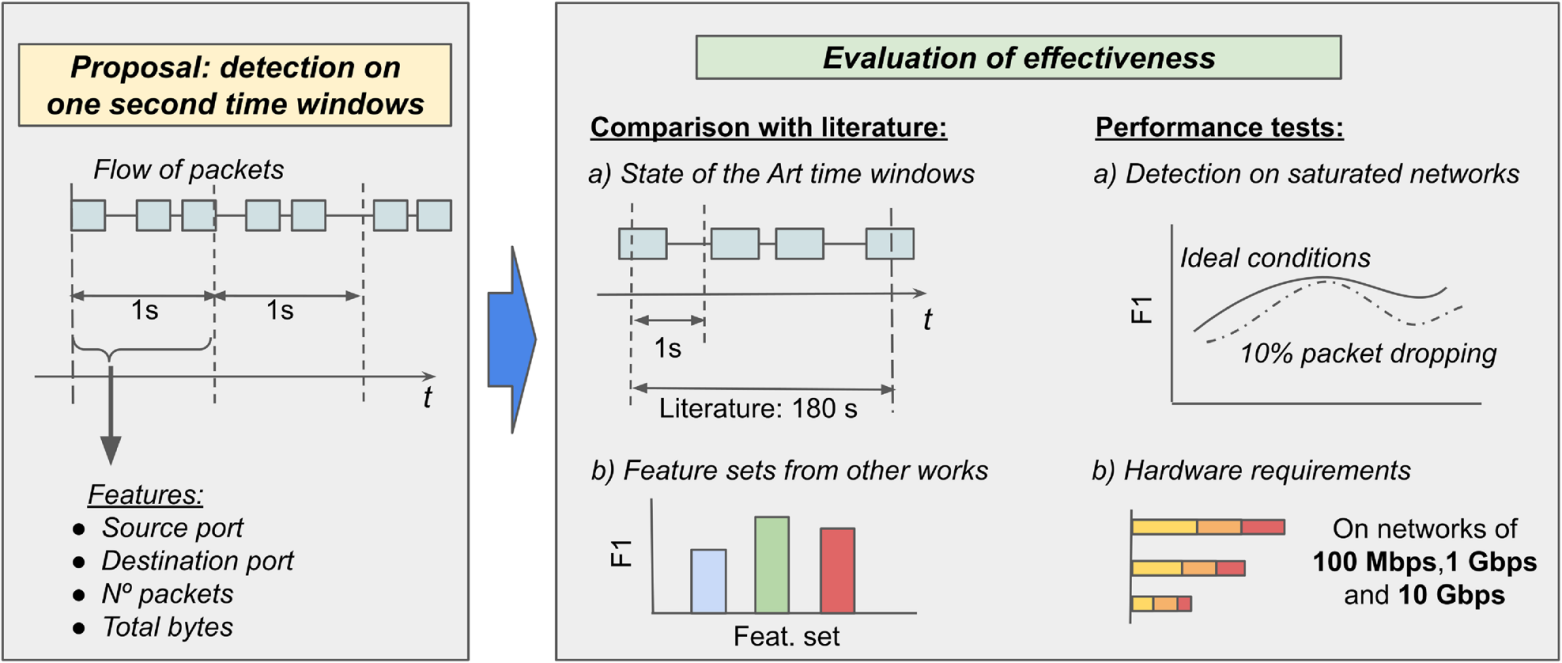
Visualization of the contributions presented in this work. We proposed an efficient classifier of network traffic capable of working on time windows of one second and yield the result in the following 1 to 2 seconds. Our model uses four features: the two port numbers (i.e., destination and source), the number of packets, and the number of bytes transmitted. First, we compared the F1 score of our model working on time windows of one second against time windows of 180 s, as recommended by the literature. Then, we compared our model against three other models from the state-of-the-art. Next, we assessed the capability of our model to work on saturated networks, and finally we measured the hardware requirements to attain a real-time detection using our model.

Finally, we compared our model to other three state-of-the-art approaches that could also be implemented to use time windows of one second, and we assessed that our approach was an order of magnitude faster. Figure 1 shows the graphical abstract of this paper.

The rest of the paper is structured as follows: “State of the Art” Section presents the state-of-the-art on network traffic analysis under real-time restrictions. “Methodology” Section explains the methods used in this work, while “Experimentation and Results” Section presents the experiments carried out in this work and the dataset used, as well as the obtained results. The conclusions of this work and future lines of work are explained in “Conclusions” Section.

## State of the art

Several works have tackled the problem of optimizing botnet detection from different perspectives and considering different aspects of the traffic. While our work is focused on the traffic generated in communications between bots or with the botmaster – the device that controls the bots –, other noteworthy approaches^18–20^ focused on detecting suspicious DNS requests: in this case, the bots include a string generator or Domain Generator Algorithm (DGA) and the server of the botmaster is hidden behind one or more of the domains that would be generated. Specifically, the bots would try to connect with the botmaster by making DNS requests with each of the generated domains, until one works. Recently, Yin et al.^21^ developed ConnSpoiler, a system optimized to detect this kind of DNS requests on IoT networks. The detector was based on the Threshold Random Walk (TRW) algorithm, and it was tested over a private dataset made with traces from a Chinese ISP network, which offered Internet services for the education, research, scientific and technical communities, relevant government departments and Hi-Tech Enterprises. The system ran over a single CPU of 2.00 GHz, and it only needed $3.3\%$ of its capacity per device to be monitored, with a false positive rate of about $0.13\%$. Highnam et al.^22^ developed the *Bilbo the“bagging”* model, which combined two neural networks, a CNN and a LSTM, to determine whether a URL is legitimate or generated with a DGA. In their experimentation on four hours of real traffic, Bilbo discovered five potential botnets that commercial tools did not warn about. Finally, DNS-based approaches can be complemented with other methods, creating frameworks such as BotDet^23^, which combines a DGA detector with other three modules based on blacklists for TOR servers and malicious IPs and SSL certificates.

Regarding approaches that analyze the inter-bot communications, one of them is to analyze traffic from various locations at the same time. This is specially relevant in distributed environments. One example is BotGuard^24^, a lightweight analyzer for SDN. It gathers the traffic data from switches in the network, which is then analyzed with a detection engine based on a Convex Lens Imaging graph (CLI-graph). According to their experiments on self-generated traffic from an SDN and using the computational power of an Intel i5 CPU, their system is able to detect botnet activity with an accuracy higher than $90\%$ and a delay lower than 56 ms.

In IoT communications, Borges et al.^25^ developed an anomaly detector that only required to measure the number of packets sent by a device in time windows of just 0.1 s. In more detail, the concatenation of these measures makes a time series that is firstly transformed in a series of *symbolic patterns* through an ordinal pattern transformation, which is then analyzed by the detector in search for anomalous patterns. According to their results on the N-BaIoT dataset, they achieved accuracies between $98.5\%$ and $100\%$ when they used time series of length $m \ge 400$. As a limitation, they reported that constructing and processing the time series required at least 1 minute before performing the detection. After that, the detection time was 24 ms in the best case and 40 ms in the worst case scenario.

Singh et al.^26^ developed a scalable implementation using open-source tools such as Hadoop^27^, Hive^28^ and Mahout^29^ to process high volumes of data. The traffic classification algorithm was Random Forest, and it was tested with CAIDA datasets^30^. This approach was able to extract the features of 1 GB pcap files in 51 s, and, thus, it could process high bandwidths of data in quasi-real-time with a 5 to 30 s of delay, while attaining an accuracy of $99.7\%$.

It is also possible to develop models that work on generic traffic^31^ such as BotMiner^32^. A frequent strategy to optimize these detectors is the selection of the best features: it reduces the characteristics to extract from the data and also the complexity of the classifier. In^33^ the features are selected using the Gini Importance metric, and the classification is made with a Random Forest (RF) classifier. Over the well-known CTU-13 dataset^34^, this approach achieved an accuracy of $90\%$ and the RF was able to classify 204, 711 samples in 27.1 seconds.

The approach used in this work is based on reducing the set of packets considered as a flow, so the feature calculation can be triggered earlier. The most usual way to achieve this is through using time windows: In^35^, Kirubavathi and Anitha use a mixture of data from the ISOT^36^ and CAIDA datasets as well as traces collected from a private setup of infected machines, to test different sizes of time-windows in botnet detection (i.e., from 60 to 300 seconds). They found that the highest performance is achieved on the time windows of 180 s, with a Naive Bayes (NB) classifier that obtained $99\%$ of accuracy and $0.02\%$ of false positive rate. Besides, Zhao et al.^37^ also studied the feasibility of detecting botnets using a limited number of packets via time windows. For their work they used two datasets from The Honeynet Project^38^ for the botnet traces, and other two datasets from the Traffic Lab at Ericsson Research and from the Lawrence Berkeley National Laboratory (LBNL) for non-malicious traces. Using a Decision Tree (DT) classifier, they found that the false positive rate (FPR) and the true positive rate (TPR) started becoming stable in time windows of 60 s, and that the best performance was achieved at 180 seconds. In this case, the DT achieved a TPR of over $90\%$ and an FPR under $5\%$. Moreover, Mai and Noh^39^ experimented with the size of the time windows in botnet detection, finding that too many flows were generated if the lengths of the time windows are shorter than 150 seconds, and that if the length was greater than 300 seconds, the accuracy was reduced. The experiments were carried out on the ISOT dataset with an ensemble model that combined k-means and DT, and the peak accuracy obtained was $99.4\%$, being relatively stable around this value if the time windows were inside the range between 150 and 300 seconds. In the same line, a recent work by Nguyen et al.^40^ proposed a collaborative detector that used features based on both network traffic and on computer processes, which also divided the activity on time windows. Their model also peaked in performance using time windows of 180 s.

Finally, other works used time windows shorter than 180 s by combining the flow analysis with other techniques. For example the multilayer framework proposed by Ibrahim et al.^41^ combined two modules to detect Command and Control (C &C) servers, which denote the external servers that control botnets and give them commands. The first Filtering module clusters the traffic into known and unknown classes using a semi-supervised Kmeans; and the second one is a “Detecting C &C Server Module” that works on the unknown class. This framework used time windows of one second and achieved a $92\%$ F1-score on the CTU-13 dataset. However, due to the clustering process the detection is not made in real-time.

## Methodology

### One-second time window

Network devices communicate through packets, and a chain of packets interchanged by two devices is called a traffic flow. It is possible to extract features from a flow and classify the nature of the communication. Although using all the packets of a connection from start to end could give the most information, it is not strictly necessary. It is possible to just use the subset of packets that fall inside a time window and calculate the desired features using them. As we already pointed out, some researchers looked for an optimal time window to detect botnet activity and settled on 180 s^35,37,39,40^. However, their research focused on attaining the best detection rate rather than spotting botnets in a small amount of time. Our goal is to detect botnets in real-time while assuming an acceptable loss in the accuracy as a trade-off.

Our approach is composed by a first module that divides the incoming traffic into time windows of one second, and inside each time window, it separates the flows, i.e., each independent communication between two devices. These flows are distinguishable by the pair of IPs and the pair of used ports. Then, for each 1s flow, the module extracts four features:

- The source port number.
- The destination port number.
- The number of packets inside the time window.
- The total bytes transmitted in that second.

The selection of these four features obeys the restriction of “being fast to calculate”, since the core design of our approach is to be able to work with large bandwidths without adding delays to the detection, at the expense of a higher detection performance. Therefore, we discarded features that require calculating means, standard deviations, or a deeper look into the packet structure. We also chose the model to work with time windows of one second since it is intended to work as an Intrusion Detection System (IDS), and one second is enough to alert humans to start a response. Next, the second module performs the classification using these features. Following the above-mentioned time restriction, we chose the classifier Decision Tree (DT), since it is fast in the decision^17^, and because it is possible to quickly train a new DT with traffic traces of new or modified botnets.

With respect to the features we have used, it is true that the source and destination ports could be circumvented by a botnet by modifying the ports and using common ones. However, this is already done by the botnets Bunitu or Miuref, which are detected by our approach.

### F1 score on saturated networks

It is unavoidable that real networks experience saturation, and thus some packets are dropped by the routers or other network devices. We have taken this situation into account and compared the performance of our proposal in both ideal conditions and in saturated conditions. For this comparison, the model was always trained on ideal conditions, i.e., without packet dropping. Then, the model was validated (i) over traffic without dropped packets, and also (ii) over traffic where each packet was subjected to a probability of 10% of being dropped. If the packet was dropped, then it was not used to extract the features.

We selected a 10% packet drop as it is usually considered as the maximum tolerable packet loss to consider a network operative^42,43^. Hence, if this rate was higher, then human administrators should be alerted, independently of the type of traffic in the network. Commercial IDS are usually not adapted for large bandwidths or saturated networks. For example, in bandwidths of 10 Gbps, Suricata^44^ showed a 74.3% false positive rate and a 16.7% false negative rate, while dropping 8% of UDP packets and 9% of TCP packets itself due to its analysis^45^. The reason behind is that they offer a more detailed analysis and can be configured to do more tasks, and thus the comparison of their method with our model would be unfair.

### Performance evaluation

Once we assessed the feasibility of our proposal, we compared its performance against other works that follow a flow based classification, and whose flows can be redefined to fit in time windows of one second. All those works were based on DT for classification and differed on the features used, as shown in Table 1. We prepared a second version of the model of Gahelot and Dayal^46^, since the original version showed a bad performance by relying on the IPs of the packets as features, as it is discussed in Subsection 4.4. The HAIS model is the DT presented in our previous work^12^.

**Table 1:** Features used by the different models tested with time windows of one second.

Feature	Ours	Zhao et al.^37^	Gahelot and Dayal^46^	Modified Gahelot and Dayal^46^	Velasco-Mata et al. (HAIS)^12^
Source IP	–	–	$\checkmark$	–	–
Destination IP	–	–	$\checkmark$	–	–
Source Port	$\checkmark$	–	–	–	$\checkmark$
Destination Port	$\checkmark$	–	$\checkmark$	$\checkmark$	$\checkmark$
Average packet length	–	–	$\checkmark$	$\checkmark$	$\checkmark$
Variance of packet lengths	–	$\checkmark$	–	–	$\checkmark$
Total null packets	–	–	$\checkmark$	$\checkmark$	–
Length of first packet	–	$\checkmark$	$\checkmark$	$\checkmark$	–
Protocol	–	–	$\checkmark$	$\checkmark$	–
Duration	–	–	$\checkmark$	$\checkmark$	–
Total bytes transmitted	$\checkmark$	–	$\checkmark$	$\checkmark$	$\checkmark$
Number of packets transmitted	$\checkmark$	$\checkmark$	$\checkmark$	$\checkmark$	$\checkmark$
Number of flows per total flows	–	$\checkmark$	–	–	–
Average interval time between packets	–	–	–	–	$\checkmark$
Variance of interval times between packets	–	–	–	–	$\checkmark$
Aver. device’s response time after receiving a packet	–	–	-	–	$\checkmark$
Var. device’s response time after receiving a packet	–	–	–	–	$\checkmark$
Number of SYN flags	–	–	–	–	$\checkmark$
Average speed of packet transmission	–	–	–	–	$\checkmark$
Variance of speed of packet transmission	–	–	–	–	$\checkmark$

Finally, we measured the time required by our proposal to calculate the features and classify one second of traffic assuming three different bandwidths: 100 Mbps, 1 Gbps and 10 Gbps. This evaluation was made in a single thread fashion, and we used this data to give insights on the hardware requirements needed to achieve a real-time performance through parallelization.

## Experimentation and results

### Dataset and preprocessing

The data used in the experiments came from the combination of traffic captures in PCAP format from two sources. On one hand, the traces from the botnets Donbot, Murlo, NSIS, Neris, Rbot, Sogou and Virut were obtained from the malware captures of the CTU-13 dataset^34^. In particular, the sixth capture’s botnet of CTU-13 was originally named “Menti”, but at the time of writing this paper, the downloadable file labels it as “Donbot”. On the other hand, we collected the traces corresponding to normal traffic and to the botnets Bunitu, Miuref and NotPetya from publicly available captures from the Stratosphere IPS Project^47^, which are the most recent publicly available captures to the best of our knowledge. We selected these three botnets because other works reported that they were relatively hard to classify^48,49^. In particular, Bunitu and Miuref make use of common ports such as 80 and 443 that are also used by regular traffic, which makes these botnets specially challenging to be detected by classifiers that use information from the ports, such as ours. To extract the traffic traces from the botnets, we used all the available captures for these traffic classes at the end of 2020 at the Stratosphere IPS Project. Regarding the normal traffic, the traces were extracted from 12 captures from uninfected computers browsing the Internet, also published by the Stratosphere IPS Project. The dataset is composed by joining the data from these traffic captures.

We used pyshark^50^ to preprocess the data from the PCAP format, extracting from each packet the necessary information to calculate the features listed in Table 1, specifically its timestamp, length (i.e., size), protocol, source and destination pairs of IP address and port, and whether it carried the SYN flag. To discern between different communications we used the IP addresses and the ports. Besides, we used the timestamp to divide the traffic in time windows.

We worked with two sizes of time windows to calculate the features of each flow, i.e., the set of packets sent between two computers during that time window. This causes a different number of samples per class: for example, 20 packets can be interchanged inside a time window of 180 s, thus generating one sample, but they are not going to generate 180 samples if the time window size is one second, as many of those windows will be empty of this communication. Table 2 shows the number of samples per class if the traffic is divided either in time windows of one second or 180 seconds.

**Table 2:** Samples extracted per traffic class in the used dataset when the traffic is divided in time windows of one second or 180 s.

Class	Samples (1 s)	Samples (180 s)
Normal	1,251,570	40,954
Bunitu	62,700	1967
Donbot	7886	465
Miuref	242,667	9395
Murlo	14,558	922
NSIS	21,162	1140
Neris	383,669	14,514
NotPetya	991	35
Rbot	139,635	4639
Sogou	454	6
Virut	86,818	3880

### Detection on time windows of one second

We compared the F1 score attained by our proposal using either time windows of (i) one second or (ii) 180 seconds, as recommended by the literature^35,37,39^. The traffic was characterized using four features: the source and destination ports, the number of packets and the total bytes transmitted within the time window. The approach uses a DT with the default configuration of the Scikit-Learn framework for Python, which uses the Gini Impurity *H* to determine the quality *G* of a candidate split $\theta$ of a node *m* of $n_m$ samples^51^ as shown in Eq. (1):

1 $$\begin{aligned} G(Q_m, \theta ) = \frac{n_m^{left}}{n_m} H(Q_m^{left}(\theta )) + \frac{n_m^{right}}{n_m} H(Q_m^{right}(\theta )) \end{aligned}$$

**Figure 2:**
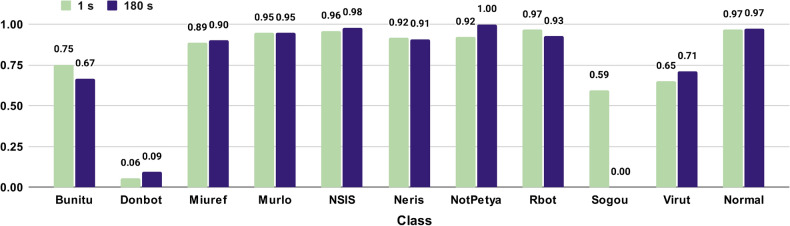
F1 scores per traffic class using time windows of either one second or 180 seconds.

Figure 2 shows the comparison between using time windows of one second or 180 seconds. These results are the mean values from a 10-fold cross-validation, with stratified folds to preserve the percentage of samples of each class. In all cases, the F1 scores achieved when using one-second windows were similar or better to those obtained when using windows of 180 seconds. The case of Sogou is especially remarkable, i.e., 0.59 in the case of 1 s and less than 0.01 in the case of 180 s. This is due to the big difference in the number of samples between the two approaches, as shown in Table 2. We have observed that Sogou established longer connections than the other botnets, and thus dividing its traffic within longer time windows implied that Sogou generated significantly fewer samples. However, NotPetya also was noticeably less represented than the other classes, but the classifier achieved a high F1 score. The reason behind this is that the traffic generated by NotPetya differs highly from the other classes, since it uses different ports. Finally, the F1 score on Donbot is exceptionally small, i.e., lower than 0.1 in both approaches. About $96\%$ the Donbot traffic and $30\%$ of Neris traffic were directed to port 25, causing that the classifier misidentified most of the Donbot traffic as Neris one. The F1 score of Neris did not suffer as much as with Donbot because it had a greater representation of samples and most of them were correctly identified.

The detection results are similar for most of the considered botnets, whether using time windows of 1 or 180 seconds. The features used were the source and destination ports, which do not change with the size of the window, and the total number of packets and total number of bytes transmitted inside the window, which do change. This suggest that the behavior of these botnets is regular over time^52^, which is expected since botnets are programs that aim to be as small as possible to avoid detection, that in turn, is contrary to have a complex code.

### Effectiveness on saturated networks

**Figure 3:**
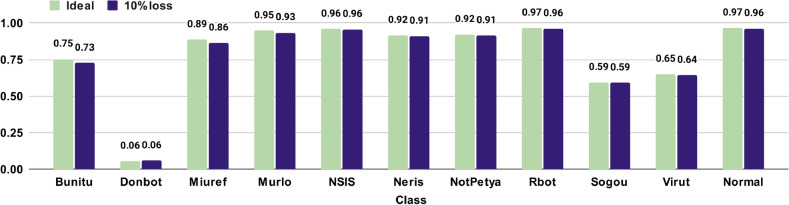
F1 scores for each traffic class on ideal conditions and on a saturated network with a packet loss probability of $10\%.$.

The next experiment compared the performance of our approach in either (i) ideal conditions or (ii) in a saturated network. In the latter case, the network traffic is reproduced in the same way as the ideal case, but each package is subjected to a probability of being dropped of $10\%$, i.e., we generate a random real number between 0 and 1 and, if it is lower than 0.1, it is removed from the raw data, so it is not processed (see “F1 scoreon saturated networks” Section). Figure 3 shows the differences in the F1 score attained for each traffic class. These results are the mean values from a stratified 10-fold cross-validation. Since our approach is based on features extracted from packets achieved in time windows of just one second, which resulted in using just one or few packets for classifying the traffic, the F1 scores did not suffer significantly from the packet losses. As previously explained in “Detection on time windows of one second” section, the low F1 score on the Donbot botnet is due to most of its traffic being misclassified as Neris traffic.

### Evaluation of performance and hardware recommendations

In this Section, we compared our proposal with three works: the model of Zhao et al.^37^, which also uses four features, the model of Gahelot and Dayal^46^ and the model from one of our previous works, which we refer as HAIS^12^.

Regarding the proposal of Gahelot and Dayal, we found that it suffered from a big flaw by using the IPs as features. The IPs could be used for aggregating purposes^53^, but never as raw features since (1) they were not assigned by the botnet malware, (2) they could be masked with NATs or similar devices and (3) they could be used to identify the infected machine in the training in the same way as using the label as a feature. This means that algorithms would learn that “devices with IP A are infected, and devices with IP B are not”, so they would perform great in the same dataset but poorly in others or in a real scenario. To prove this, we also assessed a modified version of the model of Gahelot and Dayal that did not use the IPs as a feature. In this experiment, we divided the dataset into a training set with $70\%$ of the samples, preserving the percentage of samples of each class from the dataset, and a test set with the rest of the samples in a way that the IPs of the test set would not be present in the training set.

Table 3 shows the weighted F1 scores obtained by the models to compare their detection performance, and the mean time they required to calculate the features and classify a single sample. As expected, the original version of Gahelot and Dayal^46^ performed poorly by relying on the IPs as a raw feature attaining an F1 score of 0.095, while the modified version not only was slightly faster but also achieved a F1 score of 0.911. The best F1 score was achieved by our HAIS model, which used thirteen features, but it also was the slowest one. However, our new proposal attained a similar F1 score – i.e., only $2.1\%$ lower than the HAIS model – while being the fastest, analyzing a sample in a mean of 0.007 ms. This makes it more suitable for an environment where a rapid process of lot of data is necessary.

**Table 3:** Weighted F1 scores and mean time to process a sample. The traffic was processed in time windows of one second. The *modified* Gahelot and Dayal model refers to the feature vector in which we have excluded the IPs.

Model	F1 score	Mean time/sample (ms)
Zhao et al.^37^	0.882	0.095
Gahelot and Dayal^46^ (Original)	0.095	0.048
Gahelot and Dayal^46^ (Modified)	0.911	0.046
Velasco-Mata et al. (HAIS)^12^	**0.947**	0.503
Our proposal	0.926	**0.007**

Significant values are in bold.

Even though using a smaller number of features in our model increased its speed compared to other models, one possible limitation could be a potential lack of robustness. A botnet designer might try to use common ports such as 80 (HTTP) and 443 (HTTPS) to hide the communications of the bots. However, this is already the case of many of the botnets used in the experimentation: Bunitu, Miuref, Rbot and Virut do use these ports. Still, our classifier was able to differentiate the normal traffic–that includes web navigation, thus using ports 80 and 443–from these botnets with an acceptable rate. A more sophisticated botnet might also try to mimic normal traffic by emulating the size and frequency of transmission of its traffic packets. This, however, could also work against the botnet itself for two reasons. First, it would add complexity to the malware code, making it less stealthy for antivirus programs and harder to transmit due to the extra size of the program. And second, bots usually try to limit their communications to avoid being too noisy and, thus, being detected. If they tried to imitate normal traffic, which does not have this limitation and therefore transmits more data, then they would generate a noticeable amount of traffic that could be traced back to them.

Finally, we assess the hardware requirements of our proposal for different network bandwidths. We simulated three bandwidths to test the computational cost of a DT: 100 Mbps, 1 Gbps and 10 Gbps. This simulation was done by gathering real flows generated at different times into the same time window, in a way that the system receives to analyze 100 Mb, 1 Gb or 10 Gb of data each second, respectively with the tested bandwidths. The module was built using Python 3 and ran on an Intel(R) Xeon(R) E5-2630v3 (CPUs of 2.4 GHz, 3.5 GHz on Turbo mode).

**Figure 4:**
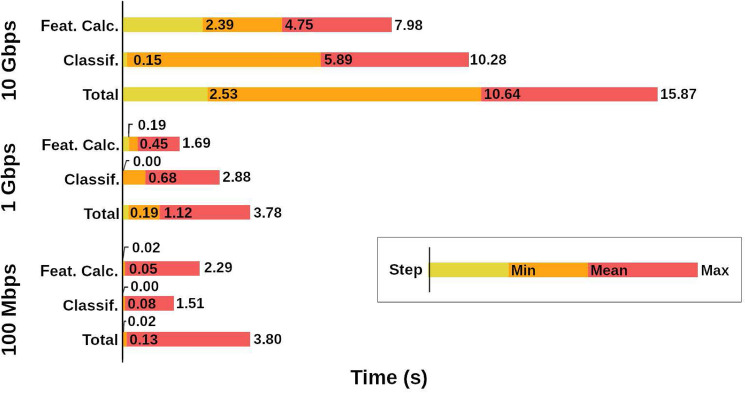
Maximum, mean and minimum time (s) required to calculate features, classify samples and the total process if the system used time windows of one second in traffic bandwidths of 100 Mbps, 1 Gbps and 10 Gbps.

Figure 4 shows the maximum, mean and minimum time required by our model to calculate the features, classify the samples and the whole process over the three bandwidths.

It is noticeable that the feature calculation took less time than the classification itself, thanks to our selected feature set, and that both 100 Mbps and 1 Gbps connections required a maximum total processing time of approximately 3.8 seconds per one second of traffic. This means that if the process was parallelized it would need at least 4 CPU cores of the same frequency (or equivalent) to classify one second of traffic in one second of processing.

In the case of 10 Gbps bandwidth, the maximum time required for analyzing one second of traffic was 15.9 s (i.e., including the features calculation and the classification), but taking into account that in the worst case the feature calculation needed 8.0 s and the classification needed 10.3 s, the safest option with parallelization would require at least 19 CPU cores at the same frequency or the equivalent.

## Conclusions

We presented a real-time approach to detect botnets, based on a Decision Tree and a small set of four features that do not require complex calculations. With the goal of detecting botnets as fast as possible, ideally at the moment in which they start working, we designed our model so that it worked on time windows of just one second, in contrast to the 180 seconds previously recommended in the literature^35,37,39,40^. We believe that it will make it possible to make a fast detection in an instantaneous fashion. We calculated the F1 score of our approach on time windows of both one second and 180 seconds. The results of this experiment showed that the score did not regress significantly by using windows of one second, while it allows us to process the traffic and detect botnets in the next 1 to 2 seconds. This makes us claim that it is possible to detect botnet-like traffic in a real-time regime based on analyzing traffic second by second.

We also took into consideration that in real scenarios the network could be overloaded and thus a percentage of packets might be dropped by the routers or other devices due to insufficient capacity. According to^45^, popular detection tools like Suricata dropped up to $9\%$ of the packets on saturated networks. Therefore, we simulated harsh conditions where the packets had a probability of $10\%$ of being dropped and tested the performance of our approach. We found that our model’s detection capability did not suffer significantly.

Once we assessed the feasibility of our approach, we compared it with other models that could be implemented for windows of one second. We observed that our approach was the fastest, needing only 0.007 ms to process a single sample, and attaining an F1 score of 0.926, only 2 percentage points lower than the approach that got the best F1 metric in our assessment.

We also gave experimental evidence against using the IPs of the network traffic as direct features to train models. While using the IPs is useful for aggregating traffic and then calculating features, using directly the IP of a device as a feature is equivalent to using the sample’s label as a feature. In other words, the model would learn that “devices with this particular IP are infected”, but this knowledge would be worthless on other datasets or in a real environment and also would mislead the model.

With respect to the hardware requirements for our proposal, we measured the time required by the system when it only uses one CPU core of 2.4 GHz (3.5 GHz in Turbo Mode) to process network bandwidths of 100 Mbps, 1 Gbps and 10 Gbps. According to this data, when the process was parallelized we could achieve real-time detection using 4 similar CPU cores for networks of 100 Mbps and 1 Gbps, and 19 CPU cores for 10 Gbps bandwidths.

In future works, we will work on testing the proposed model on other botnets, creating a knowledge database of botnets that can be quickly detected with this model, and botnets that need more sophisticated techniques.

## Data Availability

The original datasets used during the current study are available in the Stratosphere Lab repository, available at https://www.stratosphereips.org/. The processed versions used in this work are available from the corresponding author on reasonable request.
